# A Few Steps Forward, A Few Steps Back

**DOI:** 10.1089/whr.2021.0013

**Published:** 2021-04-08

**Authors:** Kimberly M. Lumpkins, Patricia Turner

**Affiliations:** ^1^ Department of Surgery, University of Maryland School of Medicine, Baltimore, Maryland, USA.; ^2^ General Surgery, University of Chicago, Chicago, Illinois, USA.; ^3^ American College of Surgeons, Chicago, Illinois, USA.

It is no secret that medical training coincides with peak reproductive years for women; for those with prolonged training periods such as in surgical fields this tension is even worse. Choosing to delay childbearing until after training carries a risk of rising infertility. A physician who chooses to pursue pregnancy faces a minefield of potential obstacles, ranging from inability to take adequate parental leave or decrease an aggressive call schedule during late pregnancy, bias by colleagues and supervisors, and concerns about childcare availability—which have reached a fever pitch due to COVID-19.

In a recent survey of members of the Association of Women Surgeons, 85.6% worked an unmodified schedule until birth and 63.6% felt that this adversely affected their own health or the health of their unborn child.^1^ The lack of flexibility in graduate medical education deters many women from having children during training. Although 40% of respondents in a survey of Mayo Clinic residents reported plans to have children during training,^2^ graduate medical education programs often do not support pregnancy and parental leave programs adequately. In 2016, the American College of Obstetricians and Gynecologists (ACOG) issued a policy statement supporting paid parental leave for all residents.^3^ The statement further recommends that trainees should not be required to make up call missed due to parental leave and that residents be clearly informed about whether leave would lengthen the duration of training. Unfortunately, in a subsequent study 71% of obstetrics and gynecology program directors (PDs) were unfamiliar with the ACOG's own policy recommendation. Even more alarmingly, 82.8% of the PDs felt that becoming a parent negatively affected resident performance.^4^

In this issue, Song and colleagues^5^ (http://online.liebertpub.com/doi/10.1089/whr.2020.0046) have explored the pregnancy and fertility experiences of gynecologic oncologists, a surgical field that is unusual due to its female predominance combined with its training focus on fertility and reproduction. The authors surveyed both men and women practitioners. Two thirds of women respondents reported delaying childbearing as a result of their career, and more women than men reported that their career influenced their family planning. Men were more likely to have children during residency, whereas women were more likely to delay until after fellowship. Forty-five percent of respondents who experienced fertility issues kept their experience private from colleagues and 13% felt stigmatized by their struggle to conceive.

Despite progress for the past decades, this study highlights the continued pressures that physicians face when considering childbirth and fertility—even those training within a specialty focused on these issues. Perhaps it is no surprise that a survey respondent described minimizing pregnancy and fertility issues during training: “I didn't talk about it…[and] made sure as much as possible that it didn't impact how I functioned or was perceived as a trainee.” This stigma disproportionately impacts women, contributing to the wall of obstacles that prevent truly equal representation in medicine. The ACOG parental leave policy is one example of tangible progress in recent years, but policy statements without widespread adoption and support are meaningless. Cultural change in medicine is slow. It is the responsibility of all of us to create a culture that supports and values families for all.

## Abbreviations Used

ACOG: American College of Obstetricians and Gynecologists
PD: program director
